# Infliximab-Associated Acneiform Eruption in a Patient With Inflammatory Bowel Disease

**DOI:** 10.7759/cureus.18213

**Published:** 2021-09-23

**Authors:** Usman Feroze Khatana, Ali Qamar, Mohammad B Ashfaq

**Affiliations:** 1 Internal Medicine, Bedford Hospital, NHS Trust, Bedford, GBR; 2 Internal Medicine, Kettering Hospital, NHS Trust, Kettering, GBR; 3 Internal Medicine, Kettering General Hospital, Kettering, GBR

**Keywords:** infilximab, inflammatory bowel disease, vedoluzimab

## Abstract

Biologic agents are increasingly used for many autoimmune and inflammatory conditions, as they are both steroid sparing and can potentially induce and maintain remission. Notably tumor necrosis factor (TNF) alpha antagonists are particularly useful in inflammatory bowel disease (IBD) such as Crohn’s disease (CD) and ulcerative colitis (UC).

Infliximab is a chimeric monoclonal antibody that targets TNF alpha (cytokine involved in modulation of inflammatory responses) and neutralizes its effects. As infliximab is a generic TNF alpha inhibitor, it can cause non-specific immune mediated side effects in addition to its intended therapeutic effect on the target organ (i.e., the gut in IBD).

We present a case of a gentleman developing a rare dermatological side effect of an acneiform reaction, after the use of infliximab for his CD. Monitoring anti-TNF alpha antibodies may help identify patients at a higher risk of developing adverse reactions. In addition, gut specific biologic agents (vedolizumab) may be the next preferable step in individuals with IBD who demonstrate reactions and/or intolerance to non-specific TNF alpha inhibitors.

## Introduction

Increasingly, inflammatory bowel disease (IBD) is being treated with biologic agents, to try and achieve control of flares and in many cases they are able to achieve remission. These agents are often reserved for escalation of treatment after failure of previous first line treatments (steroids, azathioprine, 6-mercaptopurine) or intolerance to these in addition to five aminosalicylates especially in ulcerative colitis (UC).

There are different types of biologic agents used in IBD, including TNF-α inhibitors namely infliximab, adalimumab, golimumab, an IL-12/23 inhibitor (ustekinumab), and an α4β7 integrin inhibitor (vedolizumab). Of these, the most commonly used agents are those that have longer safety evidence notably infliximab and adalimumab [1].

As these agents target the immune system and have variable levels of specificity to their target organ/lesions, their potential to cause a range of immune-mediated side effects is one of their main adverse risks. Listed for infliximab in particular, the most common side effects include infections, rashes, infusion reactions, hypersensitivity reactions, formation of autoantibodies, Lupus like syndrome, serum sickness, vasculitis, and exanthum [2]. The cutaneous adverse effects mentioned in the literature are generalized pruritus, maculopapular, eczematous, lichenoid, and granulomatous exanthems. Few cases of erythema multiforme and Stevens Johnson syndrome have also been reported [2].

## Case presentation

We present the case report of a 44-year-old gentleman with Crohn’s colitis, diagnosed in 2011, who was referred to Bedford Hospital by the IBD nurses due to a rash.

He is a gentleman who had previously been documented as intolerant to five aminosalicylates, azathioprine, and six-mercaptopurine (flu-like symptoms with all in addition to joint pains with the latter two). His treatment was thus escalated to infliximab in September 2012. The dosing and interval were guided by British National Formulary (BNF) and NICE (National Institute of Care and Excellence) guidance. He had a dose of 120 mg of infliximab at week 0, week 2, and week 6. This was followed by an eight-weekly regime of infliximab. The regular infusions were stopped in June 2015, on the patient’s own volition, as he felt well and asymptomatic from his IBD and thus felt he no longer needed it.

He then developed symptoms consistent with a clinical flare-up of Crohn’s in March 2019 and a sigmoidoscopy showed moderate to severely active left colonic Crohn’s disease (CD), with histology suggestive of moderate to severe active chronic proctocolitis. A multidisciplinary team (MDT) meeting was held in May 2019 and a decision was made to restart infliximab infusions.

**Figure 1:**
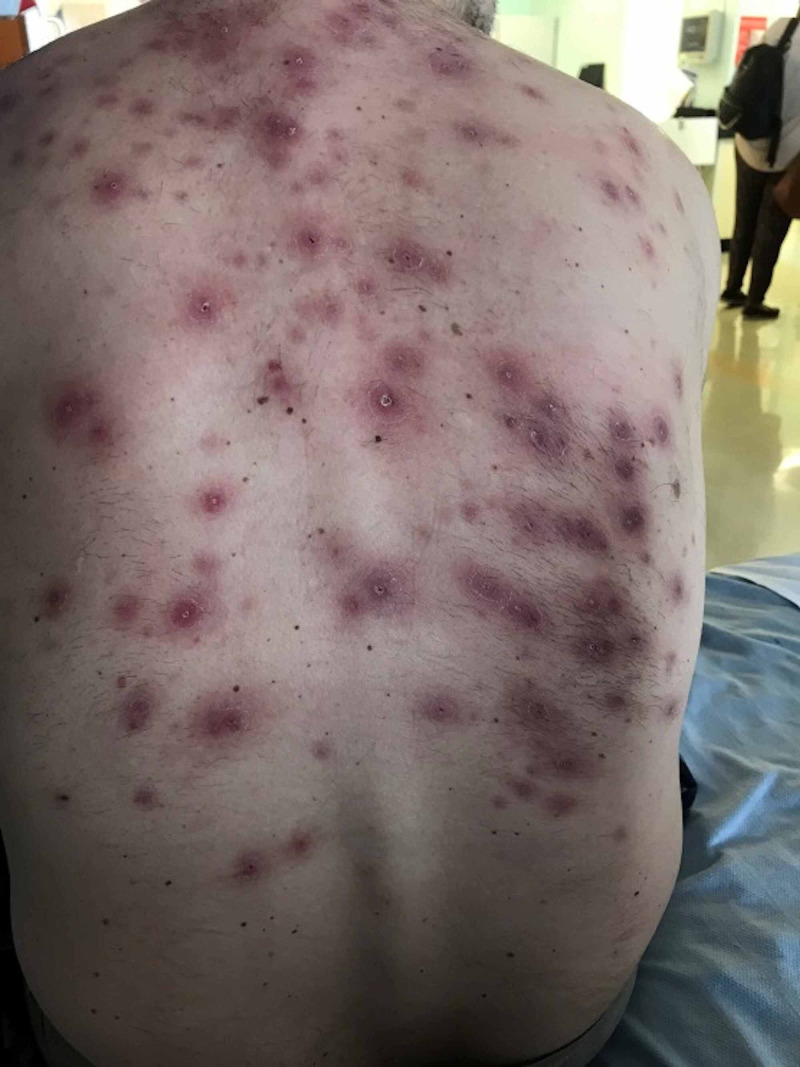
Patient's rash on presentation.

**Figure 2:**
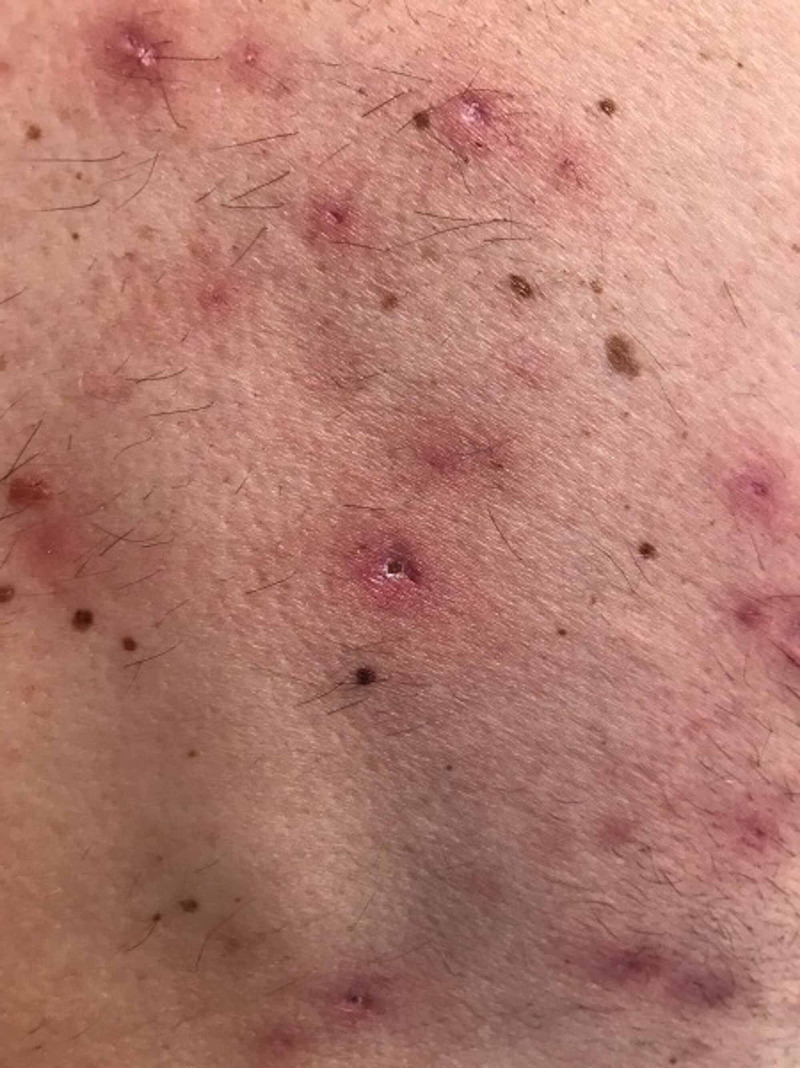
Image of the patient's skin lesions.

The offending rash then developed after receiving the first two loading doses administered at week 0 and week 2. There was no difference in dosing of infliximab as compared to his initial regime. The rash was described as acneiform eruptions on the back and neck plus eczematous lesions on elbows (Figures 1-2). The elbow lesions were initially acneiform pustular lesions shown by the pictures patient took himself. No features of psoriasis were observed. Swabs were taken which were negative and just showed skin flora.

His past medical history included no previous significant dermatological problems. During review it was noted that his bowel frequency was up to 10 times per day with a stool consistency of type 6/7 mixed with blood as per the Bristol Stool Chart; he also had three deep mouth ulcers, which were limiting his oral intake.

During his admission he had a dermatology review and they felt that this was an acneiform eruption, secondary to his anti-TNF alpha therapy. No biopsies were taken by the dermatology team. In literature the usual histopathological description given is of eosinophilic pustular folliculitis with eosinophils predominantly present within hair follicles [3].

This is a rare reaction. From our literature review, it was noted that acneiform reactions in patients treated with anti-TNFα therapy have only been reported in 11 cases (to the best of our knowledge) (Table 1) -- nine of which were with infliximab and two with adalimumab [3-6].

**Table 1:** Number of recorded acneiform eruptions, secondary to TNF-alpha inhibitors, in literature.

Number of patients with acneiform reaction	Indication for infliximab use	Reference
Six	Ankylosing spondylitis	Four patients from Sun et al. [6]; two patients from He et al. [3]
Two	Rheumatoid arthritis	Steels et al. [7]
One	Pustular psoriasis	Steels et al. [7]
Two	Crohn’s disease	He et al. [3]

**Figure 3:**
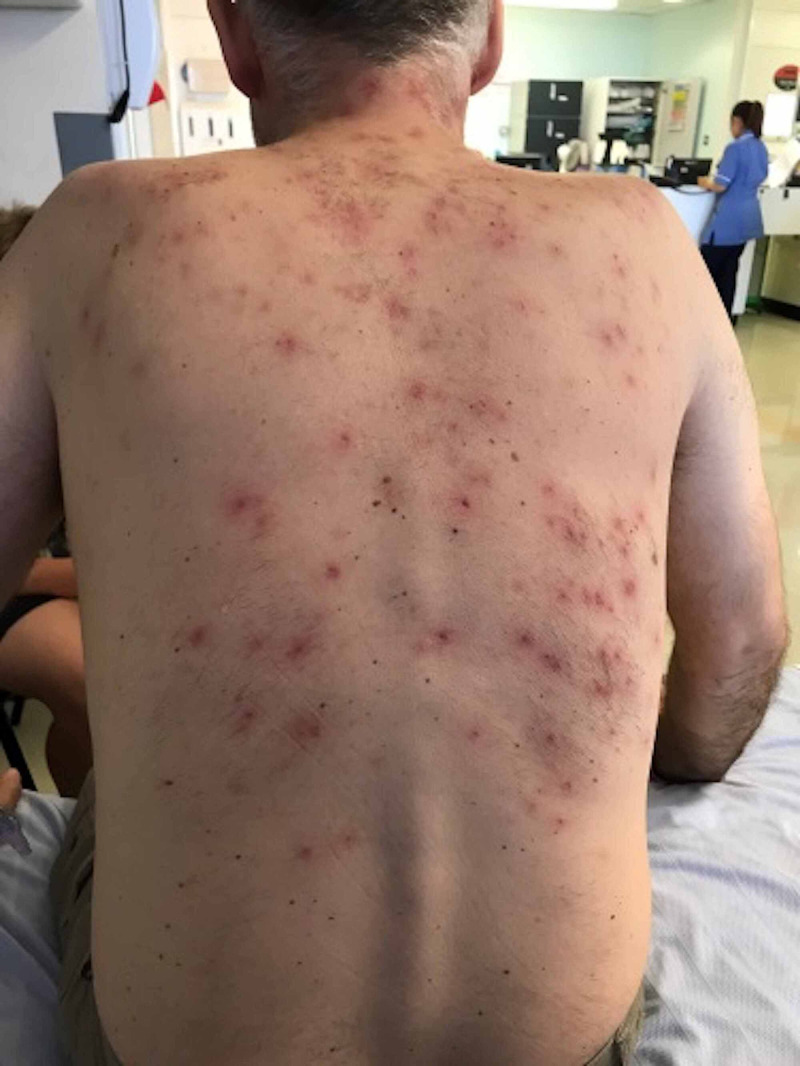
The rash gradually resolved over the course of few days.

Recommended treatment for the patient was to stop infliximab and consider acitretinoid if needed. However, our patient continued to improve without the retinoids (Figure 3).

Gastroenterology opinion was to avoid TNF alpha inhibitors in the future and the patient was switched to more gut specific biologic, in this case vedolizumab as it offers a better safety profile in terms of systemic side effects [7].

## Discussion

Although infliximab is a widely studied and used biologic agent in cases where patients do not respond to conventional treatment options for IBD, there are reported dermatological presentations associated with its use. Acneiform eruptions, however, are a rarely reported type of such reactions.

Recommendations are to test antibody levels to the drug if there is suspicion that the drug might be the offending agent. Literature review suggests that the most common time in which such acneiform eruptions are found is roughly one to two months after starting the agent. This correlates with our patient’s time of presentation. In addition, the suggestions are that the drug be discontinued and potentially other TNF alpha inhibitors be avoided in the future. Newer more target organ specific biologic agents are required which have fewer systemic side effects. In our case vedolizumab proved a useful alternative.

We felt obliged to write up this case report to add to the body of evidence with regard to association of this rather rare dermatological adverse effect with anti-TNF treatment to increase its awareness.

## Conclusions

Antibody levels in patients with prior therapy with infliximab should be done prior to restarting therapy to aid in predicting any risk of adverse reactions and hence considering therapy with alternate biologic agents. Vedolizumab has been shown to be more target organ specific and hence has lower risk of systemic side effects.
